# The value of core lab stress echocardiography interpretations: observations from the ISCHEMIA Trial

**DOI:** 10.1186/s12947-015-0043-2

**Published:** 2015-12-18

**Authors:** Akihisa Kataoka, Marielle Scherrer-Crosbie, Roxy Senior, Gilbert Gosselin, Denis Phaneuf, Gabriela Guzman, Gian Perna, Alfonso Lara, Sasko Kedev, Andrea Mortara, Mohammad El-Hajjar, Leslee J. Shaw, Harmony R. Reynolds, Michael H. Picard

**Affiliations:** Cardiac Ultrasound Laboratory, Massachusetts General Hospital and Harvard Medical School, 55 Fruit Street Yawkey 5E, Boston, MA 02114 USA; Department of Cardiovascular Medicine, Division of Cardiology, National Heart and Lung Institute, Imperial College, Sydney Street, London, SW3 6NP UK; Department of Medicine, Montreal Heart Institute, 5000 Belanger St, Montreal, QC H1T 1C8 Canada; Department of Cardiology Research, CSSS du Sud de Lanaudiere, 911 Montee des Pionniers, Terrebonne, QC J6V 2H2 Canada; Departamento de Cardiologia, Hospital Universitario La Paz, Paseo de la Castellana, 261, Madrid, 28046 Spain; Cardiologia Riabilitativa e Preventiva, Ospedali Riuniti of Ancona, via Conca 71, Ancona, Marche 60020 Italy; Hospital de Especialidades, Centro Medico Nacional ‘La Raza’, CRC IMSS, Gabriel Mancera 222 Col. del Valle, Benito Juarez, DF 3100 Mexico; Interventional Cardiology, University Clinic of Cardiology, Vodnjanska 17, Skopje, Macedonia; Department of Clinical Cardiology and Heart Failure, Policlinico de Monza, Via Amati 111, Monza, MB 20900 Italy; Division of Cardiology, Internal Medicine, Albany Medical College and Stratton VA Medical Center, 47 New Scotland Ave. MC 44, Albany, NY 12208 USA; Department of Medicine, Division of Cardiology, Emory University School of Medicine, 1462 Clifton Road, Atlanta, GA 30322 USA; Department of Medicine, Division of Cardiology, New York University Langone Medical Center, 423 East 23rd Street 15150 N, New York, NY 10010 USA

**Keywords:** Stress echocardiography, ISCHEMIA Trial, Interpretation variability, Left ventricular myocardial ischemia, Image quality

## Abstract

**Background:**

Stress echocardiography (SE) is dependent on subjective interpretations. As a prelude to the International Study of Comparative Health Effectiveness with Medical and Invasive Approaches (ISCHEMIA) Trial, potential sites were required to submit two SE, one with moderate or severe left ventricular (LV) myocardial ischemia and one with mild ischemia. We evaluated the concordance of site and core lab interpretations.

**Methods:**

Eighty-one SE were submitted from 41 international sites. Ischemia was classified by the number of new or worsening segmental LV wall motion abnormalities (WMA): none, mild (1 or 2) or moderate or severe (3 or more) by the sites and the core lab.

**Results:**

Core lab classified 6 SE as no ischemia, 35 mild and 40 moderate or greater. There was agreement between the site and core in 66 of 81 total cases (81 %, weighted kappa coefficient [K] =0.635). Agreement was similar for SE type - 24 of 30 exercise (80 %, K = 0.571) vs. 41 of 49 pharmacologic (84 %, K = 0.685). The agreement between poor or fair image quality (27 of 36 cases, 75 %, K = 0.492) was not as good as for the good or excellent image quality cases (39 of 45 cases, 87 %, K = 0.755). Differences in concordance were noted for degree of ischemia with the majority of discordant interpretations (87 %) occurring in patients with no or mild LV myocardial ischemia.

**Conclusions:**

While site SE interpretations are largely concordant with core lab interpretations, this appears dependent on image quality and the extent of WMA. Thus core lab interpretations remain important in clinical trials where consistency of interpretation across a range of cases is critical.

**Trial registration:**

ClinicalTrials.gov NCT01471522

## Background

Stress echocardiography (SE) is widely used for diagnosis, risk stratification, and prognosis of patients with known or suspected coronary artery disease and has reasonable sensitivity and specificity for clinical decision making [1, 2]. Since SE relies on the subjective assessment of wall motion abnormality (WMA) there is the potential for differences in interpretation by different readers to influence its generalizability. In fact, SE interpretations at individual institutions may be influenced by local standards and conventions and thus if reviewed at other institutions might result in different diagnoses especially in borderline cases [3]. Guidelines have been developed in order to reduce inter-reader variability [4]. However, there is still the potential for less agreement in SE readings when compared beyond a single institution, such as when utilized in multicenter studies, and this risk is a major justification for the use of a centralized core laboratory interpretation. We utilized SE from sites participating in an international multicenter trial in order to examine the concordance between site and core laboratory SE interpretation and to identify factors which might influence this concordance.

## Methods

### Subject population

SE exams submitted in the pre-enrollment site certification phase of the **I**nternational **S**tudy of **C**omparative **H**ealth **E**ffectiveness with **M**edical and **I**nvasive **A**pproaches (ISCHEMIA) Trial were studied. The ISCHEMIA Trial is a randomized study comparing an initial invasive strategy of cardiac catheterization, revascularization, and optimal medical therapy with a conservative strategy of optimal medical therapy alone among stable patients with at least moderate myocardial ischemia [5, 6]. As a prelude to this trial, participating sites were encouraged to submit stress imaging studies to the core laboratories. The SE could be any mode of stress (exercise or pharmacologic) and could demonstrate any degree of ischemia from none to severe, as the goals of this phase were to determine if the digital submissions could be transmitted smoothly and viewed on the core lab workstation, and also to assess if the site could differentiate varying degrees of ischemia. Ninety two digital SEs in quad screen format were electronically submitted to the echocardiography core laboratory at the Massachusetts General Hospital from April 2012 to April 2013. Of these 92 cases, 11 were excluded for technical reasons (very poor image quality or non-compatible format), leaving 81 cases submitted from 41 sites from 12 countries (Australia, Australia, Canada, Germany, Hungary, Italy, Korea, Macedonia, Poland, Spain, United Kingdom and United States). Demographic and clinical data were not provided to the core lab. The mode of stress was exercise in 30, pharmacologic in 49 (dobutamine, dipyridamole or adenosine) and unknown in 2. Fourteen of the cases used contrast agents for left ventricular (LV) opacification. The institutional review board of Massachusetts General Hospital approved the study protocol.

### Core laboratory assessments

Two experienced core lab echocardiographers who were blinded to the site interpretations interpreted the SE together for segmental WMAs. Standard interpretations were performed assessing each segment at baseline and peak stress using a modified 17 segment model with segment 17, the apical cap, excluded [7]. Significant WMA was defined as stress-induced severe hypokinesis or akinesis and the degree of LV myocardial ischemia was classified by the number of segments with new or worsening stress-induced segmental WMAs. Moderate or greater LV myocardial ischemia was defined as associated with an approximately 5 % per year rate of MI or death. Based on literature review and expert consensus, this was determined as occurring when at least 3 segments, developed significant WMA during SE [8]. Thus, mild ischemia was defined as one or two segments with stress-induced WMAs.

Core lab determination of image quality was based on adequacy of LV border definition and classified as excellent, good, fair, and poor. Excellent image quality had complete LV endocardial border definition. Good image quality had visualization of 88 - 99 % of the endocardial borders (14 or more segments). Fair image quality had visualization of 70 - 88 % of the LV endocardial borders (11–13 segments). Poor image quality had visualization of less than 70 % LV endocardial border.

### Statistical analysis

Concordance between site interpretation and core lab interpretations was examined in total and as a function of mode of stress, degree of myocardial ischemia, image quality, and the site’s geographic location. All categorical variables are presented as proportions. All analyses were performed using MedCalc software ver. 12.7.1.0 (MedCalc Software, Ostend, Belgium). Comparisons of concordance was assessed by Chi-square testing and weighted kappa coefficients (K). The value of K was graded as following: 0 to less than 0.4 was poor agreement; equal to or greater than 0.4 to less than 0.6 was moderate agreement; equal to or greater than 0.6 to less than 0.8 was good agreement; and equal to or greater than 0.8 to 1.0 was excellent agreement [9]. A *p*-value of less than 0.05 was considered statistically significant.

## Results

As seen in Table 1, there was agreement between the site and core lab interpretations in 66 of the 81 SE cases (81.4 %). Kappa was 0.635 and considered good agreement. From this table it is seen that in 13 of the 15 discrepancies the site over-interpreted the extent of ischemia compared to the core lab.

**Table 1:** Agreement of LV myocardial ischemia in a total of 81 cases

		MGH Echo core lab		
		No ischemia	Mild	Moderate or severe
Site	No ischemia	0	0	0
	Mild	4	28	2
	Moderate or severe	2	7	38
		K = 0.635		

*Abbreviation*: K, weighted kappa coefficients

### Concordance by stress mode

Table 2 shows the agreements as a function of the method of stress. There was concordance of interpretation in 24 of the 30 exercise SE cases (80.0 %) and 41 of the 49 pharmacologic SE cases (83.6 %). The agreement for exercise SE was considered moderate (K = 0.571) and good (K = 0.685) for the pharmacologic mode of stress. In addition, there were no significant statistical differences in concordance by Chi-square test (Table 3).

**Table 2:** Agreement of LV myocardial ischemia by stress type (exercise and pharmacological)

		MGH Echo core lab		
	*Exercise * *(N = 30)*	No ischemia	Mild	Moderate or severe
Site	No ischemia	0	0	0
	Mild	1	8	1
	Moderate or severe	1	3	16
		K = 0.571		
	*Pharmacological * *(N = 49)*			
	No ischemia	0	0	0
	Mild	3	20	1
	Moderate or severe	1	3	21
		K = 0.685		

*Abbreviation*: K, weighted kappa coefficients

**Table 3:** Comparisons of concordance in stress type (exercise vs pharmacological stress)

	Concordant read (*n* = 65)	Discordant read (*N* = 14)	χ^2^ *p*-value
Exercise stress (*N* = 30)	24	6	
Pharmacological stress	41	8	
(*N* = 49)			
			*P* = 0.91

### Concordance as a function of image quality

Table 4 shows the agreements stratified by image quality. There was agreement in 27 of the 36 cases with poor or fair image quality (75.0 %) and in 39 of 45 cases with the good or excellent image quality (86.6 %). The Κappa coefficient was 0.492 for the poor or fair image quality cases (moderate agreement) and 0.755 for the good or excellent image quality cases (good agreement). However, there were no significant statistical differences in concordance by Chi-square test (Table 5).

**Table 4:** Agreement of LV myocardial ischemia by image quality (poor or fair and good or excellent)

		MGH Echo core lab		
	*Poor or Fair * *(N = 36)*	No ischemia	Mild	Moderate or severe
Site	No ischemia	0	0	0
	Mild	2	9	1
	Moderate or severe	2	4	18
		K = 0.492		
	*Good or Excellent * *(N = 45)*			
	No ischemia	0	0	0
	Mild	2	19	1
	Moderate or severe	0	3	20
		K = 0.755		

*Abbreviation*: K, weighted kappa coefficients

**Table 5:** Comparisons of concordance in image quality (poor or fair vs good or excellent)

	Concordant read (*n* = 66)	Discordant read (*N* = 15)	χ^2^ *p*-value
Poor or Fair	27	9	
Image quality (*N* = 36)			
Good or Excellent	39	6	
Image quality (*N* = 45)			
			*P* = 0.29

### Concordance by degree of LV myocardial ischemia

Table 6 shows comparisons of concordance by degree of LV myocardial ischemia. Significant differences in concordance were noted based on the degree of ischemia. Thirteen of the 15 discordant interpretations were in patients with no or mild LV myocardial ischemia according to the core lab (86.6 %).

**Table 6:** Comparisons of concordance in degree of LV myocardial ischemia (no or mild vs moderate or severe)

	Concordant read (*n* = 66)	Discordant read (*N* = 15)	χ^2^ *p*-value
No or mild ischemia by core lab read (*N* = 41)	28	13	
Moderate or severe ischemia by core lab read (*N* = 40)	38	2	
			*P* < 0.01

### Role of contrast agents

Echocardiographic contrast agents were used in only a small number of the cases. Even though there was concordant interpretations in 13 of the 14 contrast cases, when these 14 cases were excluded from the analysis there was no significant differences in the results most likely due to the small sample size.

## Discussion

While SE is an important method to diagnose coronary artery disease, it is based on the subjective assessment of changes in LV WMA. Thus there is the potential for variability in interpretation amongst readers. In a prelude to the multicenter ISCHEMIA Trial, we examined the degree of agreement between enrolling site and core lab SE interpretations of cases that were representative of varying degrees of myocardial ischemia. We found that while agreement in aggregate is good, there are variables which are associated with lower degrees of inter-reader agreement. These include the degree of myocardial ischemia and the image quality. Such discordances highlight the importance of the use of a core lab in multicenter trials.

Specifically, there was agreement between local site and core lab in the interpretation of the degree of myocardial ischemia in 81 % of the cases. In the cases where there was disagreement, the majority (87 %) of these were determined by the core lab to have no or mild myocardial ischemia (defined as 0, 1 or 2 positive segments) but were interpreted as more extensive ischemia by the local site. Thus while one might have hypothesized that a major source of discrepant interpretations would be when small regions of stress induced WMAs are missed, in fact the major source of discrepancy was a tendency for local interpretations to over-estimate the extent of stress induced WMA. One can speculate that this may reflect a cognitive bias as the site interpreter might have been influenced by the clinical information about the patient, exercise performance, symptoms during the test or the stress ECG to interpret the stress echocardiographic wall motion to reflect more extensive ischemia than was present. The core lab interpretation was immune to such bias as the core lab did not have access to clinical or stress test data. An alternative explanation for our findings of more disagreement when myocardial ischemia is absent or mild during SE is that larger extent of, or more severe degrees of, WMA are easier to appreciate and thus the agreement would be better in these cases. Previous studies of inter-institutional observer agreement of SE have also found better agreement when the coronary artery disease is more extensive [4, 10, 11].

It is not surprising that 60 % of the disagreements occurred in cases where images were graded as fair or poor due to reduced visualization of left ventricular endocardium. Hoffman and colleagues previously identified image quality as an important factor influencing inter-observer variability in the interpretation of dobutamine stress echocardiograms [4]. Interestingly, even though image quality has improved in the 17 years since that report, the findings remain similar. Our study also extends those findings by examining exercise SE. The Kappa statistic was better for pharmacologic SE than for exercise SE. This reduced precision in agreement with exercise stress echocardiography may reflect the fact that imaging can be more challenging with exercise especially in these beating rapidly immediately after peak exercise than when the patient remains supine for the entire pharmacologic stress test. Since the sample size for exercise stress echo was smaller than for pharmacologic stress, this difference should be taken with caution.

Prior studies that have examined this issue typically have utilized single centers [12] or involved multiple experienced centers [3, 4]. Our study extends the observations to a more “real world” experience utilizing active clinical programs from around the world with various levels of experience.

While variability in discrimination of different degrees of ischemia may not be as important for the diagnosis of coronary artery disease (that is, establishing the presence or absence of any coronary disease), it does have implications for assessment of prognosis or for trial enrollment. For example, the ischemia entry criterion for the ISCHEMIA Trial is the presence of moderate or greater myocardial ischemia on stress testing. Our observations suggest that without core lab oversight, patients with less than moderate ischemia might be enrolled, and this could importantly affect the results. While the Clinical Outcomes Utilizing Revascularization and Aggressive Drug Evaluation (COURAGE) Trial did not show a benefit of revascularization to reduce major adverse cardiovascular events compared to optimal medical therapy in patients with stable coronary artery disease, a substudy demonstrated that the greatest reduction in ischemia as measured by myocardial perfusion imaging occurred in those with moderate to severe ischemia who underwent percutaneous revascularization [13, 14]. Analysis of outcomes based on the degree of ischemia was limited by small sample size. The ISCHEMIA Trial aims to more clearly define the role of an invasive strategy of routine angiography and complete revascularization in stable coronary artery disease patients with moderate or greater myocardial ischemia. Other clinical studies have shown that the extent and severity of myocardial ischemia is well correlated with prognosis, again highlighting the importance of accurate determination of not just the presence but also the severity of myocardial ischemia [15]. Our observations highlight the importance of a core lab not biased by the knowledge of the details of the patient and stress test in order to assure proper composition of the trial population.

The value of core lab interpretations has also been demonstrated in the interpretation of electrocardiograms in patients with acute coronary syndromes [16, 17]. These studies taken with ours show that the advantages of core laboratory interpretations include standardized assessments (especially when confounding variables are present), lack of bias from other clinical data, and low intra- and inter-observer variability.

Our study differs from prior studies examining inter-observer variability in SE interpretation at expert centers in that technologic advances have occurred and were incorporated into our cases. While prior studies included a high proportion of videotape assessments, all of our assessments were on digital images with harmonic imaging that were formatted for side by side rest and stress comparisons [3]. More recent updates to the original studies by Hoffman confirm that technologic enhancements such as digital image processing and harmonic imaging lead to better SE interpretation agreements [18]. Our data suggest that at least part of this improvement is due to the improvements that result in image quality.

While there may be different equipment, stress protocols, scanning techniques, image acquisition protocols and levels of expertise at different sites around the world, our data did not demonstrate a difference in concordance as a function of this geographic location.

Interpretation of stress echocardiograms is challenging and prior studies have shown the value of educational initiatives [19]. An added benefit of core lab interpretations is that the information can be passed back to the enrolling site and improve subsequent quality and interpretations.

### Limitations

There are several limitations in this study. First, due to the restrictions of the pilot phase of this trial, we did not have access to patient demographic data and thus we cannot assess if such characteristics would influence concordance. On the other hand, this enabled the core lab to remain free of bias. Also we did not have access to information about the site readers. Their years of echocardiography experience might explain differences in concordance. In fact, prior studies suggest that specific training and experience in SE is critical for accurate interpretations [20, 21]. In this actively enrolling trial, we do not yet have access to the coronary anatomy by cardiac CT or coronary angiography and thus are unable to assess concordance as a function of the location and severity of coronary artery disease. It is expected that use of contrast agents to enhance LV endocardial border delineation would improve image quality and reduce inter-observer interpretation variability. However, contrast was used in only 14 cases in this data set and so we were unable to assess its benefit. There were differences in the numbers of pharmacologic and exercise stress cases that composed our population. While the sample sizes of each population were sufficient for analysis, it is possible that this difference might contribute to the differences noted by modality. Lastly, SE in this trial did not incorporate new technologies such as strain imaging which would provide quantitative parameters that might influence concordance.

## Conclusions

We sought to assess the value of core lab interpretation in a real world experience involving multiple enrolling sites from multiple locations around the world with multiple levels of experience. While site SE interpretations are comparable to core lab interpretations in many cases, discrepancies occur in up to 20 %. The majority of the discrepancies result from local site over-estimation of the extent of ischemia. The major factors associated with discordance of interpretation are the extent of inducible ischemia and image quality. Thus, interpretations by experienced and expert core laboratories remain important in clinical trials where consistency of interpretation across a range of cases is critical.

## Abbreviation

SE: Stress echocardiography
WMA: Wall motion abnormality
ISCHEMIA: International study of comparative health effectiveness with medical and invasive approaches
LV: Left ventricular
K: Weighted kappa coefficients
